# Prediction model for best focus, power, and spherical aberration of the cornea: Raytracing on a large dataset of OCT data

**DOI:** 10.1371/journal.pone.0247048

**Published:** 2021-02-22

**Authors:** Achim Langenbucher, Nóra Szentmáry, Johannes Weisensee, Jascha Wendelstein, Alan Cayless, Rupert Menapace, Peter Hoffmann

**Affiliations:** 1 Department of Experimental Ophthalmology, Saarland University, Homburg/Saar, Germany; 2 Dr. Rolf M. Schwiete Center for Limbal Stem Cell and Aniridia Research, Saarland University, Homburg/Saar, Germany; 3 Department of Ophthalmology, Semmelweis-University, Budapest, Hungary; 4 Department of Ophthalmology, Johannes Kepler University Linz, Linz, Austria; 5 School of Physical Sciences, The Open University, Milton Keynes, United Kingdom; 6 Department of Ophthalmology, Vienna University, Vienna, Austria; 7 Augen- und Laserklinik Castrop-Rauxel, Castrop-Rauxel, Germany; University of Toronto, CANADA

## Abstract

**Purpose:**

To analyse corneal power based on a large optical coherence tomography dataset using raytracing, and to evaluate corneal power with respect to the corneal front apex plane for different definitions of best focus.

**Methods:**

A large OCT dataset (10,218 eyes of 8,430 patients) from the Casia 2 (Tomey, Japan) was post-processed in MATLAB (MathWorks, USA). Using radius of curvature, corneal front and back surface asphericity, central corneal thickness, and pupil size (aperture) a bundle of rays was traced through the cornea. Various best focus definitions were tested: a) minimum wavefront error, b) root mean squared ray scatter, c) mean absolute ray scatter, and d) total spot diameter. All 4 target optimisation criteria were tested with each best focus plane. With the best-fit keratometer index the difference of corneal power and keratometric power was evaluated using a multivariate linear model.

**Results:**

The mean corneal powers for a/b/c/d were 43.02±1.61/42.92±1.58/42.91±1.58/42.94±1.59 dpt respectively. The root mean squared deviations of corneal power from keratometric power (n_K_ = 1.3317/1.3309/1.3308/1.3311 for a/b/c/d) were 0.308/0.185/0.171/0.209 dpt. With the multivariate linear model the respective RMS error was reduced to 0.110/0.052/0.043/0.065 dpt (R² = 0.872/0.921/0.935/0.904).

**Conclusions:**

Raytracing improves on linear Gaussian optics by considering the asphericity of both refracting surfaces and using Snell’s law of refraction in preference to paraxial simplifications. However, there is no unique definition of best focus, and therefore the calculated corneal power varies depending on the definition of best focus. The multivariate linear model enabled more precise estimation of corneal power compared to the simple keratometer equation.

## Background

In most clinical situations dioptric corneal power is not really decisive and does not have any diagnostic or therapeutic consequence [1]. However, corneal power does have a high impact in refractive surgery and cataract surgery, and clinicians rely on accurate values for procedures such as the determination of an appropriate replacement lens power during cataract surgery, implantation of an additional lens into a phakic or pseudophakic eye, or determination of an ablation profile for corneal refractive surgery. In normal eyes an overestimation / underestimation of corneal power by 1 dioptre significantly affects the required power of a replacement lens during cataract surgery, resulting in an underestimation / overestimation of around 1.5 dioptres [2].

Currently, corneal power is most commonly derived from curvature measurement of the corneal front surface using a manual or automatic keratometer or topographer. The radius of curvature is measured in the mid-periphery at a distance of around 1.5 to 2.0 mm from the corneal apex, and radius of curvature is converted to corneal power using a keratometer index [3]. In the last decade more and more tomographers have been launched to the market with the ability to measure height data of the corneal front and back surface as well as corneal thickness (CCT). But in most cases derivation of corneal power has not changed as a result of these new measurement options [1].

Both corneal surfaces can be approximated to good accuracy by quadric surfaces. For stigmatic surfaces, a 2-axis ellipsoid characterized by a central radius of curvature and asphericity is sufficient [4]. In the case of astigmatism, a fit with a 3-axis ellipsoid is used, characterised by: 2 radii of curvature (in both cardinal meridians), an asphericity, and the orientation of the ellipsoid [5]. In the most general case, a biconic surface fit could be used to extract the radii of curvature and asphericity in both cardinal meridians together with the orientation of the steep or flat meridian [6]. The fewer degrees of freedom used to describe the parametric surface, the more robust the parameters extracted from the fit.

Converting corneal front surface radius of curvature (R_f_) to corneal power using a keratometer index involves a simplified optical model of the cornea, in which the refractive index of cornea and aqueous humour, central corneal thickness, and the ratio of corneal front to back surface curvature (back surface radius R_b_) from a schematic model eye are used. Using these assumptions, mostly taken from the Gullstrand model eye, in the normal eye corneal power referenced to the front (keratometer index n_K_ = 1.332) or to the back surface (n_K_ = 1.3375) may be derived [7].

In reality, the ratio of corneal front to back surface curvature and corneal thickness varies individually from eye to eye, and more modern schematic model eyes incorporate a more curved back surface curved than that of the Gullstrand eye from 1909. Additionally, this conversion from corneal front surface curvature to power is based on paraxial optics (linear Gaussian optics) without properly considering the asphericity of either surface [2].

Raytracing is a standard strategy for analysing optical systems [8]. A bundle of rays is traced through the refractive surfaces using Snell’s law (instead of the paraxial simplification) and restricted by an aperture stop. This strategy considers the curvature and asphericity of both corneal surfaces (front and back surface asphericity Q_f_ and Q_b_) in addition to the individual aperture diameter (PUP, pupil size) [9]. Tracing rays from a point light source at a finite (e.g. 4–6 m in front of the eye) or infinite distance, we evaluate the best focus position. But there are complementary definitions of best focus: e.g. the plane with the lowest wavefront (WF) error, the plane with the lowest root mean squared (RMS) scatter, the lowest mean absolute (MA) scatter of rays, or the smallest total spot diameter (TSD).

The objective of this paper is to

- use 2D raytracing based on a rotationally symmetric optical model of the cornea defined with central curvature (R_f_ and R_b_) and asphericity (Q_f_ and Q_b_) of the corneal front and back surface and to evaluate the best focus,
- derive best focus in terms of minimising WF error, RMS scatter, MA scatter, and TSD,
- describe corneal spherical aberration (SA), mean WF error, RMS scatter, MA scatter, and TSD for all 4 focal planes,
- calculate corneal power from the best focus position with respect to the corneal front surface as required for lens power calculation for cataract surgery.
- provide multivariate linear models for corneal power as a function of R_f_, Q_f_, R_b_, Q_b_, CCT and PUP in a large dataset of > 10,000 measurements from an OCT device.

## Methods

### Measurement data

This retrospective study included 10,218 eyes of 8430 patients examined between January 2019 and July 2020 at Augenklinik Castrop-Rauxel. All data are from a cataract population, obtained during pre-cataract examination. The 10,218 datasets from the Casia 2 (Tomey, Nagoya, Japan) were batch exported in standard .csv format and imported to MATLAB (MathWorks, Natick, USA, Version 2019b) for subsequent data analysis. Incomplete datasets were filtered out prior to exporting the data. From this data set, we used the central corneal front surface radius R_f_, the central corneal back surface radius R_b_, corneal eccentricity of the corneal front and back surface both derived in the central 6 mm zone, central corneal thickness CCT, and projected (visible) pupil size. Corneal eccentricity was converted to corneal asphericity, and quadric surfaces were defined for the corneal front and back surface based on central radius of curvature and asphericity. The sag z of these quadric surfaces is defined by:

$$z-z_{0}=\frac{R_{f,b}\cdot r^{2}}{1+\sqrt{1-Q_{f,b}\cdot R_{f,b}\cdot r^{2}}}$$

Where z_0_ refers to the axial position of the surface apex, R_f,b_ to the central radius of curvature of the front and back surface, and Q_f,b_ to the asphericity of the front and back surfaces respectively.

### Raytracing scheme

For the cornea we assumed a centred optical system without tilt. Both surfaces were considered as quadric surfaces as described above, and the apex of the corneal front surface was considered to be located at the origin, with the apex of the back surface at z = CCT. The axial symmetry of this model means that a 2-dimensional raytracing strategy was sufficient [10, 11]. The respective refractive indices of cornea and aqueous humour were taken from the Liou & Brennan schematic model eye [12].

A collimated bundle of 1001 rays starting from a plano surface at z = 0 was projected to the corneal front surface. Rays were equally spaced in the radial direction over the projection of the individual pupil size (PUP) to the corneal front surface from–PUP/2 to PUP/2. To ensure an equidistant spacing over the pupil (area correction), rays were weighted in a quadratic manner with respect to the distance from the optical axis. For each ray we calculated the intersection with the corneal front surface, the direction of the refracted ray (within the cornea) using Snell’s law, and the intersection with the corneal back surface [8]. Again, applying Snell’s law we derived the direction of the refracted ray in the anterior chamber filled with aqueous humour.

Behind the corneal back surface we calculated the wavefront curvature and fitted a circle to obtain a preset value for the focus (centre of the circle). Starting from this centre we applied a nonlinear annealing algorithm to extract the best focus planes with:

- the minimal RMS WF error (BFP-WF),
- the minimum RMS ray scatter (BFP-RMS),
- the minimum MA ray scatter (BFP-MA),
- the minimum total spot diameter TSD (BFP-TSD).

For all 4 best focus planes we analysed the RMS wavefront error (WFE), the RMS ray scatter (RMSS), the MA ray scatter (MAS), plus the TSD, together with the Zernike coefficient for spherical aberration Z40 referenced to a central 6 mm zone at the corneal front surface plane [13–15].

From the location of the best focus planes we calculated corneal power with respect to the corneal front apex plane. Defining a best fit keratometer index for each best focus plane, we investigated the dataset of 10,218 OCT measurements to define multivariate linear models describing corneal power as functions of keratometric power (based on the best fit keratometer index) and linear correction terms for corneal front and back surface radius R_f_ and R_b_, corneal asphericity Q_f_ and Q_b_, central corneal thickness CCT and pupil size PUP.

## Results

**Fig 1:**
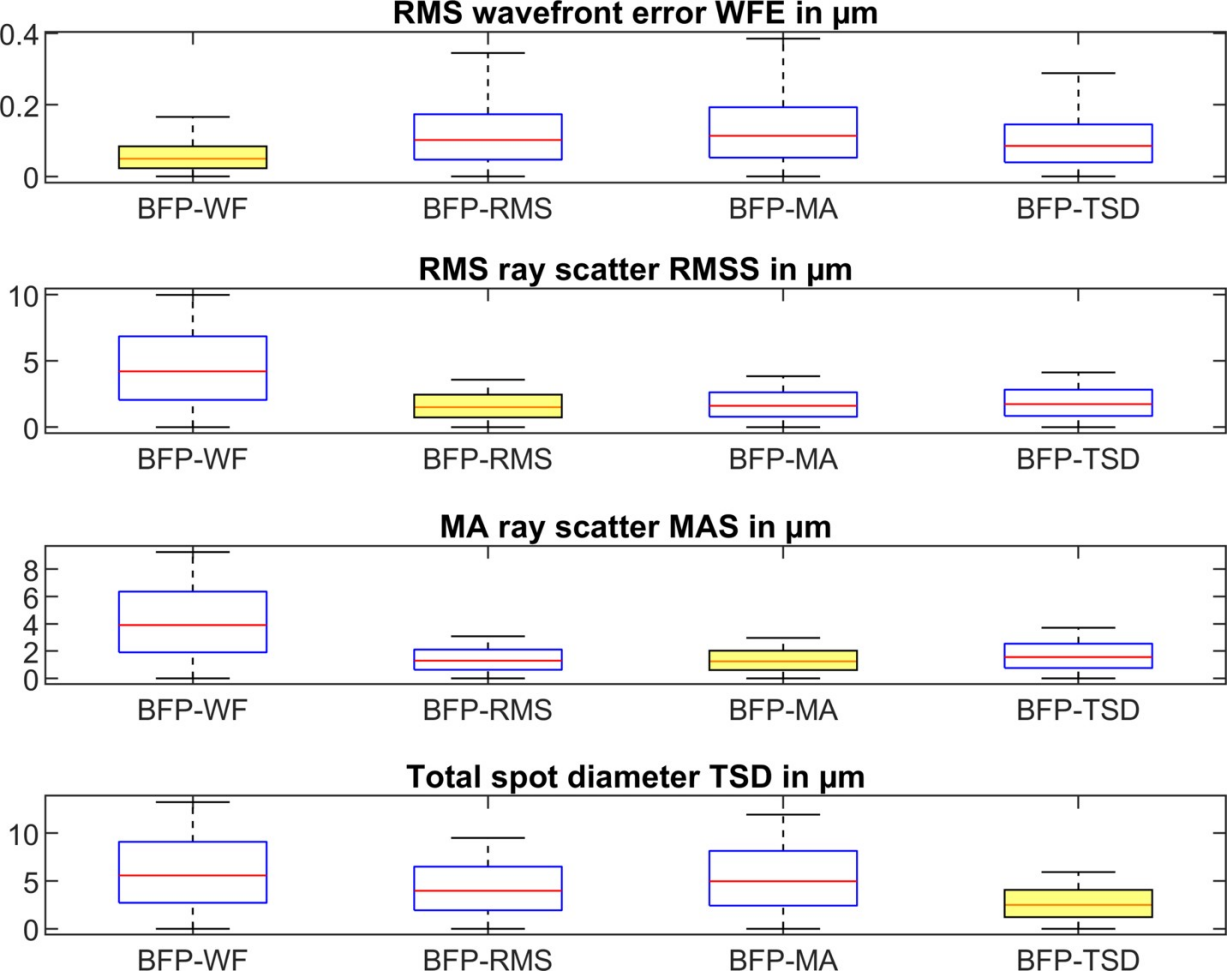
Boxplot indicating the root mean squared wavefront error WFE (row 1), the root mean squared ray scatter RMSS (row 2), the mean absolute ray scatter MAS (row 3), and the total spot size TSD (row 4). Best focal planes BFP-WF, BFP-RMS, BFP-MA, and BFP-TSD refer to the focal plane with the lowest RMS wavefront error, the lowest RMS ray scatter, the lowest mean absolute scatter, and the lowest total spot diameter respectively. This means that the optimisation was performed for the situations shown on the diagonal of the plot (row 1 / column 1 to row 4 / column 4).

The descriptive statistics of the 6 input data of R_f_, R_b_, Q_f_, Q_b_, CCT, and PUP are shown in **Table 1** in terms of mean, standard deviation, median, and 90% and 99% confidence level. **Table 2** displays the descriptive statistics for the position of the best focus planes BFP-WF, BFP-RMS, BFP-MA, and BFP-TSD alongside with the refractive power referenced to the corneal front apex plane. **Fig 1** shows the RMS wavefront error WFE (row 1), the RMS ray scatter RMSS (row 2), the mean absolute ray scatter MAS (row 3), as well as the total spot diameter TSD (row 4) for each of the 4 best focus planes BFP-WF, BFP-RMS, BFP-MA, and BFP-TSD.

**Table 1:** Descriptive statistics of the input data with mean, standard deviation (SD), median, minimum and maximum, 0.5%, 5%, 95% and 99.5% quantiles (90% and 99% confidence intervals).

N = 10,218	R_f_ in mm	Q_f_	R_b_ in mm	Q_b_	CCT in μm	PUP in mm
Mean	7.733	-0.3141	6.534	-0.007	547	4.408
SD	0.283	0.249	0.274	0.213	37	0.789
Median	7.720	-0.319	-6.530	-0.014	547	4.468
Minimum	6.770	-1.486	5.500	-0.988	395	1.528
Maximum	8.800	0.584	7.500	0.602	794	6.800
Quantile 0.5%	7.040	-0.870	5.864	-0.696	439	2.421
Quantile 5%	7.250	-0.629	6.060	-0.335	490	2.843
Quantile 95%	8.190	-0.122	6.99	0.323	609	5.360
Quantile 99.5%	8.426	0.122	7.256	0.441	646	5.822

R_f_ refers to the corneal front surface radius, Q_f_ to the corneal front surface asphericity, R_b_ to the corneal back surface radius, Q_b_ to the corneal back surface asphericity, CCT to the central corneal thickness, and PUP to the pupil size considered at the corneal plane.

**Table 2:** Descriptive statistics of the locations of the 4 best focus planes (z_F_) together with the respective corneal power CP referenced to the corneal front apex plane.

N = 10,218	BFP-WF		BFP-RMS		BFP-MA		BFP-TSD	
	z_F_ in mm	CP in dpt	z_F_ in mm	CP in dpt	z_F_ in mm	CP in dpt	z_F_ in mm	CP in dpt
Mean	31.1015	43.02	31.1706	42.92	31.1806	42.91	31.1558	42.94
SD	1.1621	1.61	1.1499	1.58	1.1489	1.58	1.1517	1.59
Median	31.0669	43.00	31.1246	42.92	31.1353	42.91	31.1153	42.94
Minimum	27.6481	37.18	27.7955	37.24	27.8135	37.26	27.7675	37.22
Maximum	35.9369	48.32	35.8709	48.06	35.8609	48.03	35.8869	48.11
Quantile 0.5%	28.2054	39.18	28.3127	39.06	28.3339	39.04	28.2812	39.09
Quantile 5%	29.2058	40.40	29.2580	40.38	29.2648	40.36	29.2462	40.39
Quantile 95%	33.0695	45.74	33.0853	45.66	33.1023	45.65	33.0766	45.68
Quantile 99.5%	34.1015	47.37	34.2030	47.19	34.2202	47.15	34.1779	47.24

The Description is given in terms of mean, standard deviation (SD), median, minimum and maximum, 0.5%, 5%, 95% and 99.5% quantiles (90% and 99% confidence intervals). Best focal planes BFP-WF, BFP-RMS, BFP-MA, and BFP-TSD refer to the focal plane with the lowest RMS wavefront error, the lowest RMS ray scatter, the lowest mean absolute scatter, and the lowest total spot diameter respectively.

**Fig 2:**
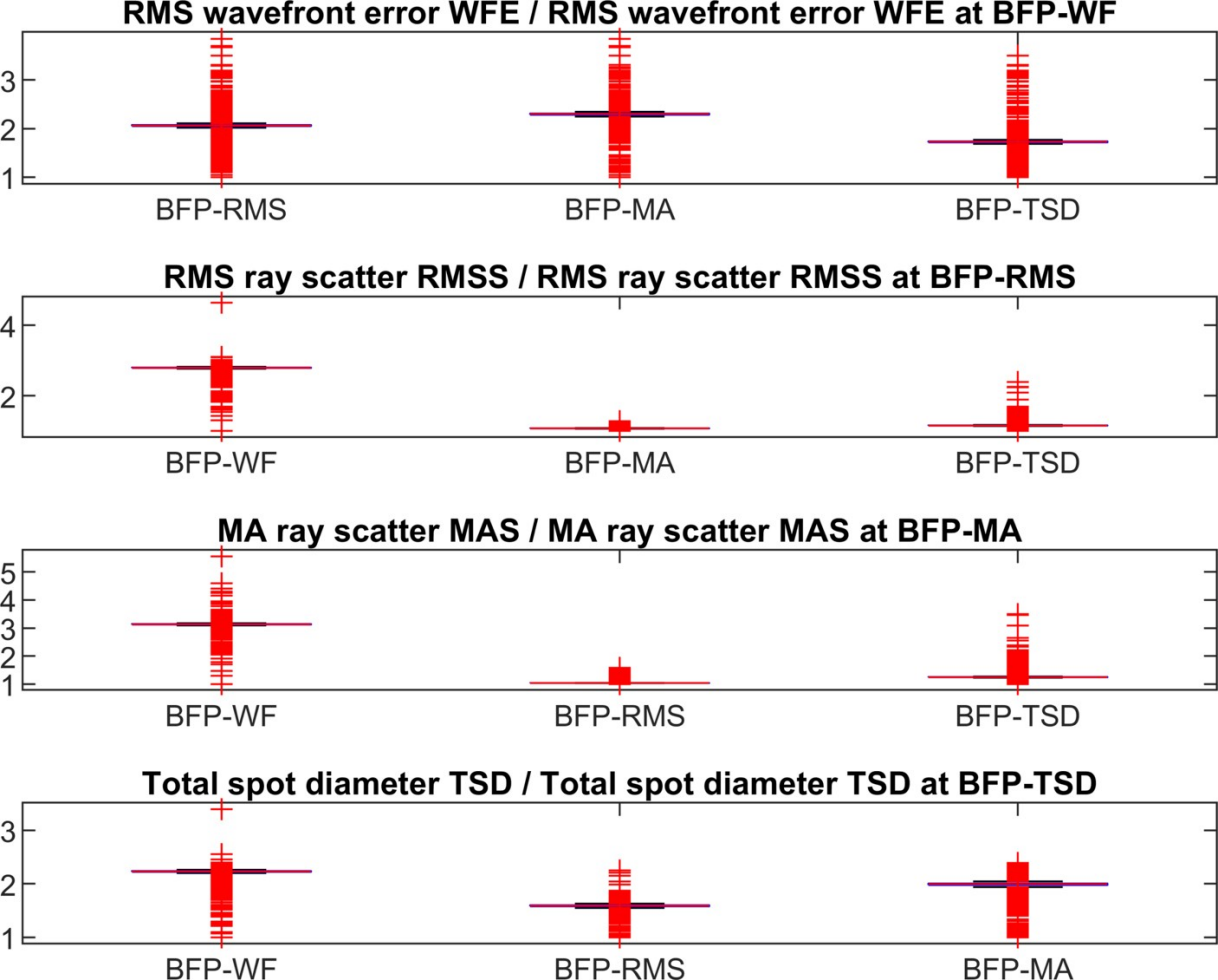
RMS wavefront error referenced to RMS wavefront error at BFP-WF, RMS ray scatter referenced to RMS ray scatter at BFP-RMS, MA ray scatter referenced to MA ray scatter at BFP-MA, and total spot diameter TSD referenced to TSD at BFP-TSD. Best focal planes BFP-WF, BFP-RMS, BFP-MA, and BFP-TSD refer to the focal plane with the lowest RMS wavefront error, the lowest RMS ray scatter, the lowest mean absolute scatter, and the lowest total spot diameter respectively.

On the diagonal of the plot we find those conditions where the target criterion fits to the respective best focal plane (e.g. BFP-WF and WFE, row 1 / column 1 or BFP-RMS and RMSS, row2 / column 2). **Fig 2** displays the ratio of RMS wavefront error to RMS wavefront error at BFP-WF, ratio of RMS ray scatter to RMS ray scatter at BFP-RMS, ratio of MA ray scatter to MA ray scatter at BFP-MA, and ratio of total spot diameter TSD to TSD at BFP-TSD.

**Table 3** provides the descriptive statistics of the corneal spherical aberration in terms of Zernike coefficient Z40 extracted from the wavefront error at corneal front apex plane for a central optical zone of 6 mm, plus the keratometer index derived from corneal power and corneal front surface radius R_f_. for BFP-WF, BFP-RMS, BFP-MA, and BFP-TSD.

**Table 3:** Descriptive statistics of the spherical aberration (Zernike coefficient Z40, considered at corneal front apex plane with a diameter of 6 mm) and the keratometer index derived from corneal power CP for the 4 best focus planes indicating lowest RMS WF error (BFP-WF), lowest RMS spot size (BFP-RMS), lowest mean absolute scatter (BFP-MA), and lowest overall spot diameter (BFP-TSD).

N = 10,218	SA (Z40) in μm for 6 mm at				Keratometer index n_K_ at			
	BFP-WF	BFP-RMS	BFP-MA	BFP-TSD4	BFP-WF	BFP-RMS	BFP-MA	BFP-TSD
Mean	0.1715	0.1744	0.1748	0.1738	1.3317	1.3309	1.3308	1.3311
SD	0.2951	0.2980	0.2984	0.2973	0.0024	0.0014	0.0013	0.0016
Median	0.1744	0.1745	0.1748	0.1741	1.3319	1.3311	1.3310	1.3313
Minimum	-1.4930	-1.4798	-1.4779	-1.4827	1.3191	1.3212	1.3215	1.3207
Maximum	1.5100	1.5376	1.5416	1.5315	1.3391	1.3361	1.3356	1.3368
Quantile 0.5%	-0.8210	-0.8170	-0.8165	-0.8179	1.3245	1.3251	1.3251	1.3252
Quantile 5%	-0.2920	-0.2927	-0.2928	-0.2926	1.3273	1.3286	1.3287	1.3284
Quantile 95%	0.6565	0.6666	0.6678	0.6644	1.3351	1.3330	1.3327	1.3334
Quantile 99.5%	1.1050	1.1232	1.1258	1.1194	1.3366	1.3341	1.3338	1.3346

The description is given in terms of mean, standard deviation (SD), median, minimum and maximum, 0.5%, 5%, 95% and 99.5% quantiles (90% and 99% confidence intervals).

**Fig 3:**
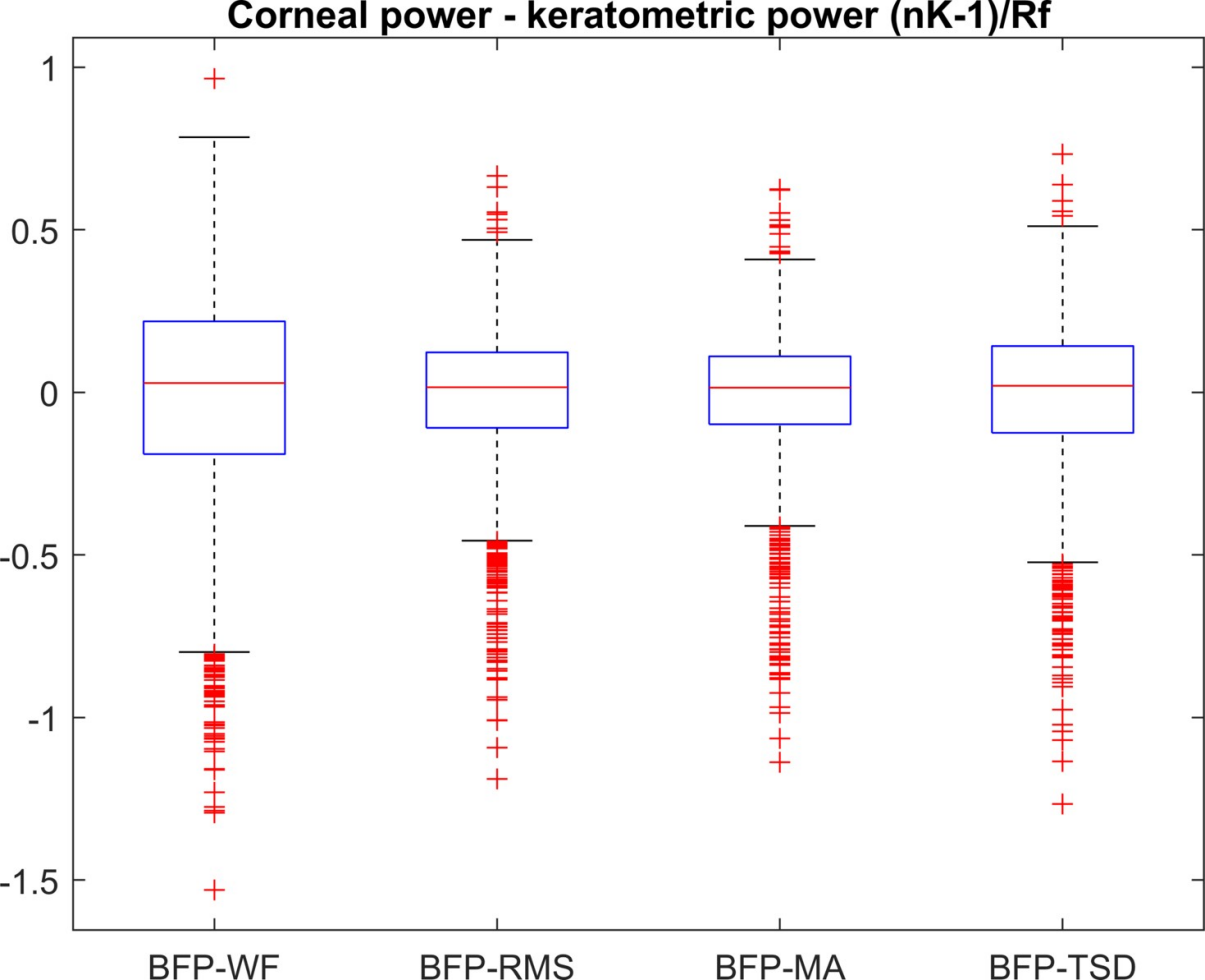
Difference of corneal power referenced to corneal front apex plane derived from best focus planes BFP-WF, BFP-RMS, BFP-MA, and BFP-TSD and keratometric power. Keratometric power was calculated using (n_K_-1)/Rf with n_K_ optimised for best focus planes 1: n_K_ = 1.3317, 2: n_K_ = 1.3309, 3: n_K_ = 1.3308, 4: n_K_ = 1.3311. Best focal planes BFP-WF, BFP-RMS, BFP-MA, and BFP-TSD refer to the focal plane with the lowest RMS wavefront error, the lowest RMS ray scatter, the lowest mean absolute scatter, and the lowest total spot diameter respectively.

The customised keratometer index for BFP-WF, BFP-RMS, BFP-MA, and BFP-TSD calculated with a least squares fit yields: n_K_ = 1.3317 forBFP-WF, n_K_ = 1.3309 for BFP-RMS, n_K_ = 1.3308 for BFP-MA, n_K_ = 1.3311 for BFP-TSD. **Fig 3** shows the difference between corneal power with respect to BFP-WF, BFP-RMS, BFP-MA, and BFP-TSD referenced to the corneal front apex plane. **Table 4** shows the respective descriptive data.

**Table 4:** Descriptive statistics of the difference between corneal power and keratometric power.

N = 10,218	Corneal power CP–keratometric power referenced to			
	BFP-WF	BFP-RMS	BFP-MA	BFP-TSD
Mean	0	0	0	0
SD	0.3079	0.1850	0.1708	0.2090
Median	0.0308	0.0163	0.0152	0.0193
Minimum	-1.5233	-1.1858	-1.1354	-1.2618
Maximum	0.9738	0.6701	0.6280	0.7373
Quantile 0.5%	-0.9226	-0.7236	-0.7168	-0.7377
Quantile 5%	-0.5639	-0.3079	-0.2761	-0.3590
Quantile 95%	0.4408	0.2652	0.2417	0.3026
Quantile 99.5%	0.6465	0.4152	0.3848	0.4589

Corneal power was derived from the best focus plane with respect to the front apex plane, and keratometer power was calculated using (n_K_-1)/R_f_ with a keratometer index specified in Table 3. The 4 best focus planes indicating lowest RMS WF error (BFP-WF), lowest RMS spot size (BFP-RMS), lowest mean absolute scatter (BFP-MA), and lowest overall spot diameter (BFP-TSD). The description is given in terms of mean, standard deviation (SD), median, minimum and maximum, 0.5%, 5%, 95% and 99.5% quantiles (90% and 99% confidence intervals).

Modelling corneal power with respect to best focus planes 1 to 4 results in the following equations:

Model 1: For BFP-WF corresponding to optimisation for root mean squared wavefront error WFE:

$$CP=\frac{1000\cdot (1.3317-1)}{R_{f}}+0.86129-0.90224\cdot R_{f}+1.7446\cdot Q_{f}+0.97129\cdot R_{b}-0.33023\cdot Q_{b}+0.29098\cdot CCT+0.062371\cdot PUP$$

Model 2: For BFP-RMS corresponding to optimisation for root mean squared ray scatter RMSS:

$$CP=\frac{1000\cdot (1.3309-1)}{R_{f}}+0.40217-0.84386\cdot R_{f}+0.81261\cdot Q_{f}+0.93662\cdot R_{b}-0.15142\cdot Q_{b}+0.32146\cdot CCT+0.029592\cdot PUP$$

Model 3: For BFP-MA corresponding to optimisation for mean absolute ray scatter MAS:

$$CP=\frac{1000\cdot (1.3308-1)}{R_{f}}+0.33451-0.83496\cdot R_{f}+0.67492\cdot Q_{f}+0.93097\cdot R_{b}-0.12361\cdot Q_{b}+0.32598\cdot CCT+0.02504\cdot PUP$$

Model 4: For BFP-TSD corresponding to optimisation for total spot diameter TSD:

$$CP=\frac{1000\cdot (1.3311-1)}{R_{f}}+0.50409-0.85727\cdot R_{f}+1.0176\cdot Q_{f}+0.94506\cdot R_{b}-0.19287\cdot Q_{b}+0.31454\cdot CCT+0.036352\cdot PUP$$

For all linear models 1 to 4, the radii of curvature R_f_ and R_b_, central corneal thickness CCT and pupil diameter PUP are defined in mm, while corneal front and back surface asphericities Q_f_ and Q_b_ are dimensionless. The root mean squared (RMS) error / R² of models 1 to 4 are 0.110 / 0.872 (model 1), 0.052 / 0.921 (model 2), 0.043 / 0.935 (model 3), and 0.065 / 0.904 (model 4). For all 4 models, the intercept and all 6 effect sizes are highly significant with p<0.001.

## Discussion

In many ophthalmological situations the dioptric corneal power is of minor relevance, without direct consequence to diagnosis or therapy [1]. However, in some situations such as planning a cataract surgery or refractive intervention at the cornea or lens, the corneal power has an extremely high impact. In modern biometry for cataract surgery, misinterpretation of corneal power is one of the most important error sources [1, 2, 8]. In spite of this, in most clinical cases ophthalmologists tend to restrict themselves to measurement of corneal front surface radii and interpret corneal power with a simplified thin lens model using a keratometer index for conversion.

However, even if the shape of the corneal front and back surface is measured with a tomographer and corneal thickness and the diameter of the aperture stop is known, raytracing does not solve all problems: even using Snell’s law, it is necessary to specify the best focus plane of the cornea. There are several options in optics to define the best focus, and in professional raytracing tools we have for instance, the options of minimising the root mean squared wavefront error, the root mean squared ray scatter, the mean absolute ray scatter, or the overall spot size at the focus plane. Besides these options, we could also optimise for the Strehl ratio or some characteristics of the modulation transfer function.

In this paper we wish to show that there is no general rule for defining best focus, and depending on the selection of a target criterion such as optimising for the RMS wavefront error WFE or RMS ray scatter RMSS we obtain different values for the best focus plane. And with different locations of the best focus plane we obtain different values for the corneal power [7, 13], for the keratometer index [3], or for spherical aberration [13–15]. We restricted the study in this paper to 4 different options for definition of the best focus plane: minimisation of the RMS wavefront error (BFP-WF), the RMS ray scatter (BFP-RMS, spot size), the mean absolute ray scatter (BFP-MA, mean spot size), and the total spot size (BFP-TSD, overall diameter of the spot). **Fig 1** shows the behaviour of these measures for all 4 best focus planes. Clearly, if we optimise e.g. BFP-WF, the RMS wavefront error WFE at BFP-WF will be lower compared to BFP-RMS, BFP-MA, or BFP-TSD. Therefore, the lowest measures are always found on the diagonal in **Fig 1**. By referencing to those optimised conditions (e.g. calculating the ratio of RMS wavefront error at best focus planes 2 to 4 to RMS wavefront error at best focus plane 1) we get a measure of robustness as shown in **Fig 2**. Based on our dataset these ratios range between 1 and 5, where 1 refers to a situation with no loss in our target criterion if we optimise for a condition A and evaluate condition B. For instance, if we consider the ratio of RMS wavefront error at BFP-RMS, BFP-MA, or BFP-TSD to RMS wavefront error at BFP-WF we see that the RMS wavefront error increases by around 100–200% (the ratio ranges between 2 and 3). It is interesting to note that if we optimise for RMS ray scatter RMSS and analyse BFP-MA or optimise for MA ray scatter MAS and analyse for BFP-RMS the ratios are more or less 1, which means that based on our dataset, both optimisation criteria could be used interchangeably.

Another interesting finding is that even if we customise the keratometer index to represent corneal power with respect to BFP-WF, BFP-RMS, BFP-MA, and BFP-TSD, we find some deviation of corneal power from keratometric power in the range -0.56 to 0.44 dpt for BFP-WF, -0.31 to 0.27 dpt for BFP-RMS, -0.28 to 0.24 dpt for BFP-MA, and -0.36 to 0.30 dpt for BFP-TSD for the 95% confidence interval (**Table 4**). This means that considering the 95% confidence interval, even in the simplified case with centred quadric surfaces, the corneal power derived with raytracing techniques deviates from the keratometric power by typically ±¼ to ±½ dioptre. This deviation has to be taken into account for lens power calculation prior to cataract surgery.

We attempted to characterise the deviation of corneal power from keratometric power (as the dependent target variable) with a multivariate linear model. As effect sizes we selected the central curvature of the corneal front and back surface, the asphericity of the corneal front and back surface, central corneal thickness, and diameter of the pupil. The root mean squared fit errors of models 1 to 4 are 0.110 / 0.052 / 0.043 / 0.065 mm respectively. Comparing these root mean squared fit error of models 1 to 4 to the root mean squared error of the deviation of corneal power minus keratometric power (0.308 / 0.185 / 0.171 / 0.209 mm), we see that with models 1 to 4 the deviation is significantly reduced. The performance of model 1 seems to be slightly lower (R² = 0.872) compared to models 2 to 4 (R² = 0.921 / 0.935 / 0.904). But overall, the deviation is reduced with the multivariate linear models to around 1/3.

**In conclusion**, corneal power can be derived from corneal front and back surface shape including central radius of curvature and asphericity, central corneal thickness, and pupil diameter. If we restrict our analysis to a centred optical system without tilt, the aspherical corneal front and back surface can easily be represented with quadric surfaces and raytracing simplified from 3D to 2D. We should be aware that, when using raytracing techniques to evaluate the focusing properties of the cornea to overcome the limitations of paraxial optics, there is no generally accepted definition regarding the best focus. Depending on the best focus definition we obtain different best focus planes and therefore different corneal powers. Keratometric power does not fully reflect the refractive properties of the cornea, and the deviation between corneal power and keratometric power ranges between ±¼ and ±½ dpt depending on the selection of the best focus criterion.

## Supporting information

S1 Data (XLSX)
